# Enabling Proteomics: The Need for an Extendable ‘Workbench’ for User-Configurable Solutions

**DOI:** 10.1002/cfg.372

**Published:** 2004-02

**Authors:** Robert J. Beynon

**Affiliations:** Department of Veterinary Preclinical Sciences University of Liverpool Crown Street Liverpool L69 7ZJ UK

## Abstract

Proteomics has the capability to generate overwhelming quantities of data in relatively short timescales, and it is not uncommon to see experimenters investing substantially more time in data analysis than in data gathering. Although several sophisticated tools for data reduction and analysis are available, they lack the flexibility to cope with increasingly innovative experimental strategies and new database resources that encode both qualitative and quantitative data. I will outline a specification of a flexible proteomics tool that could address many current bottlenecks and deficiencies.


**Comparative and Functional Genomics**
*Comp Funct Genom* 2004; **5**: 52–55.
Published online in Wiley InterScience (www.interscience.wiley.com). **DOI:** 10.1002/cfg.372

**Conference Review**

### Enabling proteomics: the need for an extendable 'workbench' for user-configurable solutions

Robert J. Beynon*
*Department of Veterinary Preclinical Sciences, University of Liverpool, Crown Street, Liverpool L69 7ZJ, UK*

**Correspondence to: Robert J. Beynon, Department of Veterinary Preclinical Sciences, University of Liverpool, Crown Street, Liverpool L69 7ZJ, UK. E-mail: r.beynon@liv.ac.uk*

Received: 13 November 2003
Revised: 18 November 2003
Accepted: 26 November 2003

### Abstract

**Proteomics has the capability to generate overwhelming quantities of data in relatively short timescales, and it is not uncommon to see experimenters investing substantially more time in data analysis than in data gathering. Although several sophisticated tools for data reduction and analysis are available, they lack the flexibility to cope with increasingly innovative experimental strategies and new database resources that encode both qualitative and quantitative data. I will outline a specification of a flexible proteomics tool that could address many current bottlenecks and deficiencies. Copyright © 2004 John Wiley & Sons, Ltd.**

**Keywords:** **proteomics; mass spectrometry; bioinformatics**

### Introduction

The science of proteomics is often seen as a large-scale exercise in systems biology. The sequential processes in proteomics are; simplification of the proteome (organelle purification, 2D gels, protein chromatography, 1D and 2D peptide chromatography); mass spectrometric analysis of the ensuing peptides (using one or more of the impressive variety of the current generation of mass spectrometers); and bioinformatic analysis of the resultant mass spectrometric data. One of the major challenges in proteomics is that of identification and quantification of every protein in a complex mixture. This goal has been a major driver in the evolution of high-throughput systems, in novel strategies for proteome simplification and in the construction of powerful bioinformatics platforms.

Yes, there are many studies in proteomics that have a rather different perspective. The focus can be constrained to a single protein or a small group of proteins. It is possible to entertain the idea of proteomics experiments without a single gene, cDNA, EST or protein sequence being present in any database. Some experimenters seek to extend proteomics by the use of clever chemical or stable isotope-based methods that greatly enhance simplification or subsequent analysis of the analyte. Whether such studies are all deserving of the title of 'proteomics' is irrelevant; they represent cutting-edge thinking in analytical and preparative protein chemistry. Imaginative as such studies are, I suggest that a major constraint on such developments is the absence of software tools to analyse the subsets and modifications to the analyte. I will present a case, based on the perspective of a protein chemist and an end-user of such software tools, for a configurable proteomics platform that allows individual experimenters to define the nature of their analyses.

### Current needs and frustrations

My research group is motivated by a range of proteomics studies that cover such diverse areas of biology as chemical communication, copper toxicity, cross-species matching and proteome dynamics. Of these studies, relatively few are driven by the need for global protein identification or by comparative proteomics. In all of the studies, I have been a little surprised by the investment of time in

Copyright © 2004 John Wiley & Sons, Ltd.

data analysis relative to the duration of the biological experiment. Whilst simple database searches (e.g. for peptide mass fingerprinting against known proteomes) are rapid and efficient, other analyses require a high degree of manual intervention and, indeed, time-consuming visual inspection of the data.

As an illustrative example, we are motivated to extend the description of a proteome to include an understanding of proteome dynamics, defined as the rates of synthesis and degradation of any protein within the proteome. Comparative proteomics is set firmly in the arena of changing proteomes, but an understanding of changes in the amount of any protein in the cell requires definition of both the rate of synthesis and the rate of breakdown. This acquires particular significance when attempts are made to correlate proteome and transcriptome data, as such studies implicitly assume that an increase in the level of protein reflects, through an increase in the mRNA level, a corresponding increase in the rate of synthesis of the proteins. However, a protein can also increase in concentration if the rate of degradation is reduced. Parameterization of proteome changes into rate of synthesis and degradation is of fundamental biological importance. Changes in rates of synthesis are very likely to correlate directly with transcriptome changes, whilst changes in degradation may reflect interactions at the substrate level, and may thus connect more immediately with the metabolome.

To analyse proteome dynamics, we use stable isotope-labelled amino acids, which are incorporated into proteins as they are synthesized *de novo*. Note that we design experiments to avoid complete labelling of the proteins, as this would eliminate any information about the relative rates of synthesis. The proteins are then separated and analysed most simply by MALDI-TOF mass spectrometry of a tryptic peptide mixture. The relative amounts of 'heavy' and 'light' peptides define the rate of replacement of that particular protein in the system. The 'heavy' and 'light' variants of the peptide are clearly identifiable, and the mass offset coincidentally informs about the number of the labelled amino acid in the peptide. However, to determine the rate of turnover we need to calculate the areas under the 'heavy' and 'light' peptide peaks. Further, we would ideally have a tool that would scan a MALDI-TOF mass spectrum, identify those peptides that exist as heavy–light pairs and calculate the rate of turnover automatically [1]. No such tool exists in any readily accessible form.

I suggest that the lack of such simple and targeted tools is an obstacle to the development of novel and imaginative approaches to the development of proteomics. It is unlikely that any one group would produce a full range of such tools, and I am persuaded by the opportunities for construction of an open, extensible platform onto which new tools and modules can be bolted as required, and offered as a service to the entire proteomics community. I refer to this concept as a 'workbench' to assist proteomics and, rather than coin another acronym (there are already too many of these in proteomics), will refer to 'workbench' throughout this article.

One of the better analogous systems that might serve as a model for the workbench is the AVS package (**http://www.avs.com**) designed for visualization of scientific data and presentation of image data. This comprises a core product onto which can be introduced task-specific tools, written by the scientific community. The tools are assembled in a modular format, with defined connectivities to the core and to other tools. Modules are assembled in a graphical environment where modules, represented as building blocks, are linked to define complete analyses. I am intrigued by the possibility that proteomics, or protein mass spectrometry, might also benefit from the availability of such an open environment into which new tools can be slotted without the added overhead of the need to write a complete application. The behaviour of each tool or module is either inflexible, performing a single invariant function, or is modified by a set of parameters that are adjusted by the user through control panels (using such visual devices as sliders, dials and check boxes) or through text commands. The workbench would have a scripting language underlying each module, and it might be possible to dispense with the visual metaphor and cast an analytical process as a script.

### A proteomics workbench

The workbench would not be intended to replace or compete with other developments for management of complete proteomics experiments, including PEDRO (**http://pedro.man.ac.uk** [2]) and the Human Proteome Organisation (HUPO) proposals (**http://psidev.sourceforge.net/** [3,4]). Rather,

Copyright © 2004 John Wiley & Sons, Ltd. 

I see it as a set of tools that at least in part can precede experimental design, encourage an analytical approach to the development of novel strategies and provide customizable modules for analysis of novel, and sometimes unique, proteomics data (Table 1).

The two major sources of data for the workbench are sequence databases and mass spectrometric data. Each covers a range of specific data types. Sequence databases can be protein sequences (SWISSPROT, TREMBL), untranslated gene or cDNA sequences (EMBL-Bank, GenBank) or EST resources (dbEST). In all instances, these datasets have utility in proteomics studies. They differ in the degree of error that they manifest (e.g. single-pass vs. multiple-pass sequencing of DNA) but, with appropriate tools, can generate a search space against which proteomics data can be matched. Typical tools that might fall under the aegis of database manipulation include extraction of a subset of sequences from multiple data sources to create a local, private database or subproteome, or generation of a summary analysis of the members of a proteome or subproteome.

**Table 1.** Some modules that might form part of a core proteomics workbench

Filters
- Selective recovery of entries from protein or DNA databases according to user-specified criteria to form a subproteome
- Filtering of a proteome according to use-defined criteria, such as presence of specific pairs of amino acids, post-translational modification sites

Processes
- Six-frame translation and recovery of all putative ORFs according to pre-defined criteria
- Scanning of mass spectra for stable isotope-labelled duplexes or multiplexes
- Summary statistics pertaining to a local subproteome
- Chemical modification strategies
- Proteolytic digestion to generate a database of fragments
- Detailed queries of proteome, subproteomes or fragments sets
- Shotgun sequencing assembly of overlapping MS/MS data
- Searching private databases using experimentally derived data (possibly externally computed using GRID-like capabilities)

Outputs
- Plot a distribution of a range of parameters, define a proteome, subproteome
- Presentation of detailed mass spectrometric coverage diagrams
- Tabulate and export database sets in text or XML format

Mass spectrometric data would be more problematical, because several instrument manufacturers use proprietary data formats that are not as readily accessible. There is a need for some intermediate mass spectrometric data format to which all instrument suppliers adhere, at least as an exportable format. This is a topic of active debate and development and which can be built upon existing programmes in analytical science (**http://psidev.sourceforge.net/ms/docs/030611_PSI_ASMS.pdf**).

### Representative modules

The modules that could be built into the workbench are limited only by the imagination of the investigator and the availability of appropriate programming skills. However, careful description of the scope and behaviour of some primitive tools should permit a hierarchical construction of task-specific tools that could be shared. Rather than devise a tool to define proteome-relevant analysis of a single database, a generic tool should be defined to operate on any global or local database. Perhaps the most appropriate way forward in defining the functionalities of the modules is by direct interaction with end-users, who will be most able to define their tasks in terms of natural language specifications.

Many workbench specifications could be initiated with three types of modules — filters, processes and outputs. The filters work on external data sources or on internally created local or private datasets, and offer rule-based simplification of the data sources. A filter might, for example, support SQL statements or allow more flexible user control via an intuitive control panel. Filters are equivalent to searches, and could be applied at an early stage or intermediate stage of any workbench application. In natural language, a filter might 'prepare a local, temporary proteome database of all proteins in TREMBL or SwissProt that are derived from chicken'. An investigator should be able to pose the task 'plot the distribution of masses of endopeptidase LysC peptides from rodent skeletal muscle, irrespective of species, and split according to whether the peptides contain no, one, two or more than two valine residues', or 'what percentage of human liver proteins have a tryptic N-terminal peptide that is between 400 Da and 4000 Da?'. These

Copyright © 2004 John Wiley & Sons, Ltd.

might seem like arcane questions, but they are the types of problem that proteome research groups are posing all of the time, and are the sort of questions that are needed to inform the development of new experimental strategies. More subtle questions, such as 'what percentage of proteins from proteome X contain post-translational site Y and what is the size distribution of those peptides?' are also common, and require an element of sequence scanning of the proteome or subproteome dataset.

For mass spectrometric data, there are a number of tasks that are not presently catered for. All high-quality mass spectra provide clear resolution of the all 12C and one 13C mass peaks at charge states up to 5. Yet, the algorithms to reduce such data to the masses of the parent peptides (sometimes referred to as 'deisotoping') is of variable effectiveness. A second task germane to our research programmes would be a simple method to scan a mass spectrum in $m/z$ space for $([M + nH]^{n+})/n$, $([M + \mathbf{pX} + nH]^{n+})/n$ pairs, where $M$ is the mass of the parent peptide, $n$ is the charge state (number of protons) and **X** is the additional mass afforded by a stable isotope-labelled amino acid that occurs **p** times in that peptide. From that scan, isotopically labelled pairs could be collated and used to enhance the processes of protein identification [5], relative quantification or even calculation of the parameters of proteome dynamics. Another specialist application might be that of 'shotgun protein sequencing', where multiple peptides, derived from digests with proteases of different primary specificities, are used to create substantial tracts of sequence information. At present, we can only perform this task by manual overlapping of interpreted peptide sequence data. There is considerable scope for a tool that builds overlaps from uninterpreted tandem mass spectrometric data and which ultimately enhances confidence in the final sequence call.

A final requirement is for high-quality data presentational tools that can create visualizations of the data using familiar and, hopefully, some novel graphical modes. However, most users will probably also require export of the data in XML or ASCII files for import into other presentational and analytical software. Any workbench should be expected to adhere to emerging standards for XML representation of mass spectrometric data.

It is not clear that such a plan would ever be realized; there may be enough interested parties to agree on the common interface and core modules that such a workbench would require. Then, the community will create the needs, from which the specification of new modules can be drawn.

### Acknowledgments

Research in my group is supported by BBSRC, NERC and the Wellcome Trust. The projects supported by these funding agencies have done much to help me identify bottlenecks and frustrations in a range of proteomics experiments.

### References

1. Pratt JM, Robertson DH, Gaskell SJ, *et al*. 2002. Stable isotope labelling *in vivo* as an aid to protein identification in peptide mass fingerprinting. *Proteomics* **2**: 157–163.
2. Taylor CF, Paton NW, Garwood KL, *et al*. 2003. A systematic approach to modelling, capturing and disseminating proteomics experimental data. *Nature Biotechnol* **21**: 247–254.
3. Orchard S, Hermjakob H, Apweiler R. 2003. The proteomics standards initiative. *Proteomics* **3**: 1374–1376.
4. Orchard S, Kersey P, Zhu W, *et al*. 2003. Progress in establishing common standards for exchanging human proteomics data. *Comp Funct Genom* **4**: 203–206.
5. Pratt JM, Petty J, Riba-Garcia I, *et al*. 2002. Dynamics of protein turnover, a missing dimension in proteomics. *Mol Cell Proteom* **1**: 579–591.

Copyright © 2004 John Wiley & Sons, Ltd. 
